# Prognostic Utility of N-terminal Pro-B-type Natriuretic Peptide (NT-proBNP) for Predicting Hospital Readmissions in Patients With Heart Failure: A Systematic Review of Clinical Evidence

**DOI:** 10.7759/cureus.85242

**Published:** 2025-06-02

**Authors:** Hafiz Shahpal Arshad, FNU Laraib, FNU Nikeeta, FNU Vandina, Aneesha Alias Gurya, Umer Farooq, Sajid Ali, Lyba Nisar, Muhammad Hamza Zamir, Farhan Akbar

**Affiliations:** 1 Internal Medicine, Quaid-E-Azam Medical College, Bahawalpur, PAK; 2 Internal Medicine, Peoples University of Medical and Health Sciences, Nawabshah, PAK; 3 Internal Medicine, Ghulam Muhammad Mahar Medical College, Sukkur, PAK; 4 Family Medicine, Jinnah Sindh Medical University, Karachi, PAK; 5 Internal Medicine, Peoples University of Medical and Health Science for Women, Nawabshah, PAK; 6 Internal Medicine, Fauji Foundation Hospital, Rawalpindi, PAK; 7 Cardiology, Rawalpindi Institute of Cardiology, Rawalpindi, PAK

**Keywords:** heart failure, hospital readmission, nt-probnp, prognostic biomarker, systematic review

## Abstract

This systematic review examines the prognostic utility of N-terminal pro-B-type natriuretic peptide (NT-proBNP) for predicting hospital readmissions in patients with heart failure (HF). HF remains a leading cause of recurrent hospitalizations, contributing to increased morbidity and healthcare burden. While NT-proBNP is widely established for diagnosis and mortality prediction in HF, its role in forecasting hospital readmission risk-particularly at varying time frames-remains unclear. A comprehensive search of PubMed, Scopus, and the Cochrane Central Register of Controlled Trials (CENTRAL) yielded 452 records, of which six studies met the inclusion criteria. These included one randomized controlled trial, one post hoc RCT analysis, and four observational or registry-based studies. The methodological quality was assessed using RoB 2.0, the NIH Quality Assessment Tool, and the Newcastle-Ottawa Scale, revealing low to moderate risk of bias.

The review found that isolated NT-proBNP values at admission showed limited predictive value, while serial measurements during hospitalization or within early post-discharge periods (e.g., 30-180 days) demonstrated stronger associations with readmission risk. Predictive variability was influenced by factors such as timing of measurement, renal function, age, and sex. Comparators, where present, varied across studies and included standard care or alternative biomarkers. Notably, current clinical guidelines lack standardized protocols for using NT-proBNP in readmission risk prediction, leading to inconsistent applications in practice. This review underscores the need for individualized interpretation and standardized measurement strategies to enhance NT-proBNP's utility in discharge planning and post-acute care of patients with HF.

## Introduction and background

Heart failure (HF) remains one of the leading causes of hospitalization and readmission worldwide, contributing significantly to healthcare costs and patient morbidity [1]. Despite advances in pharmacological and non-pharmacological management, a substantial proportion of patients experience recurrent hospitalizations, often within 30 to 180 days following discharge [2,3]. This range reflects variability across the included studies and existing literature, with some assessing short-term readmissions at 30 days and others examining medium- to long-term outcomes up to 180 days. These readmissions are not only detrimental to patients’ quality of life but also serve as indicators of suboptimal management or unresolved congestion.

Among the biomarkers explored to predict these adverse events, N-terminal pro-B-type natriuretic peptide (NT-proBNP) has garnered significant attention [4,5]. NT-proBNP is released in response to myocardial wall stress and volume overload and is widely used for the diagnosis and prognosis of HF. Mechanistically, elevated or persistently high NT-proBNP levels may reflect incomplete decongestion, ongoing hemodynamic stress, or residual myocardial dysfunction, all of which contribute to increased risk of rehospitalization. Its levels correlate closely with disease severity, ventricular filling pressures, and treatment response. Serial measurements of NT-proBNP during hospitalization or in the early post-discharge period have shown potential in identifying patients at high risk of deterioration [6,7].

However, while NT-proBNP is a well-established tool for risk stratification, its specific utility in predicting hospital readmissions remains variably supported across studies, in part due to differences in study design, measurement timing, and patient populations. The comparator across studies typically involved standard clinical assessments, which include physical examination findings, symptom burden, fluid status assessment, or echocardiographic evaluation, as well as alternative biomarkers such as BNP, red cell distribution width (RDW), or relaxin. Despite the growing body of literature, the lack of standardized NT-proBNP thresholds, heterogeneity in follow-up durations, and inconsistencies in outcome reporting have limited the ability to draw firm conclusions, thereby justifying the need for a structured systematic review.

The current review includes randomized controlled trials, prospective cohort studies, and registry-based observational studies to provide a comprehensive assessment of available evidence. Furthermore, although the population [8] comprises both acute and chronic HF patients, previous literature has not consistently differentiated the prognostic value of NT-proBNP between these subtypes. Therefore, our review addresses these groups as a composite while noting relevant subgroup findings where applicable. Given the clinical importance of reducing readmissions and improving transitional care, a systematic evaluation of NT-proBNP's predictive utility is warranted to inform individualized discharge planning and post-acute management strategies. To structure our approach, we applied the PICO framework: the Population includes patients with acute or chronic HF; the Intervention is NT-proBNP measurement as a prognostic tool; the Comparators include standard clinical assessments or alternative biomarkers; and the Outcome is hospital readmission within a defined follow-up period.

## Review

Materials and methods

Search Strategy

A comprehensive literature search was conducted in accordance with the Preferred Reporting Items for Systematic Reviews and Meta-Analyses (PRISMA) guidelines [9] to ensure methodological rigor and transparency. The search strategy was applied across multiple databases, including PubMed, Scopus, and the Cochrane Central Register of Controlled Trials (CENTRAL). Keywords and MeSH terms related to “NT-proBNP,” “heart failure,” and “readmission” were combined using Boolean operators to retrieve relevant studies. Filters were applied to include only human studies published in English within the last 20 years. To enhance the clinical applicability of the review, we restricted the results to clinical trials, prospective cohort studies, and observational studies, excluding case reports, editorials, and reviews. Duplicate records were removed, and titles and abstracts were screened for relevance, followed by full-text assessment to determine eligibility based on predefined inclusion and exclusion criteria.

Eligibility Criteria

Studies were included in this review if they evaluated the prognostic utility of NT-proBNP in predicting hospital readmissions among patients with acute or chronic HF. Eligible designs included randomized controlled trials, prospective or retrospective cohort studies, and observational studies that reported on readmission outcomes within a defined follow-up period. Only studies conducted in human subjects, published in English, and available in full text were considered. Studies were excluded if they focused solely on NT-proBNP for diagnostic or mortality outcomes without reporting data on hospital readmissions, or if they were case reports, reviews, editorials, or animal studies.

Data Extraction

Data extraction was performed independently by two reviewers using a structured data extraction form designed specifically for this review. Extracted variables included study characteristics (author, year, country, study design, sample size), patient population details, NT-proBNP measurement strategy (baseline vs. serial), intervention and comparator (if any), primary outcomes related to readmission, and key statistical findings such as hazard ratios and *P*-values. Any disagreements during data extraction were resolved through discussion or consultation with a third reviewer to ensure consistency and accuracy.

Data Analysis and Synthesis

Given the heterogeneity in study designs, NT-proBNP measurement timing, cut-off values, and reported outcomes, a meta-analysis was not conducted. Instead, a qualitative synthesis was performed, summarizing key findings across studies and identifying patterns in predictive performance. Emphasis was placed on whether NT-proBNP levels were measured at admission, during hospitalization, or post-discharge, and how these variations influenced the biomarker’s ability to predict readmission risk. Risk of bias was assessed for each study using appropriate tools based on study design, and the overall strength of evidence was narratively evaluated.

Results

Study Selection Process

**Figure 1:**
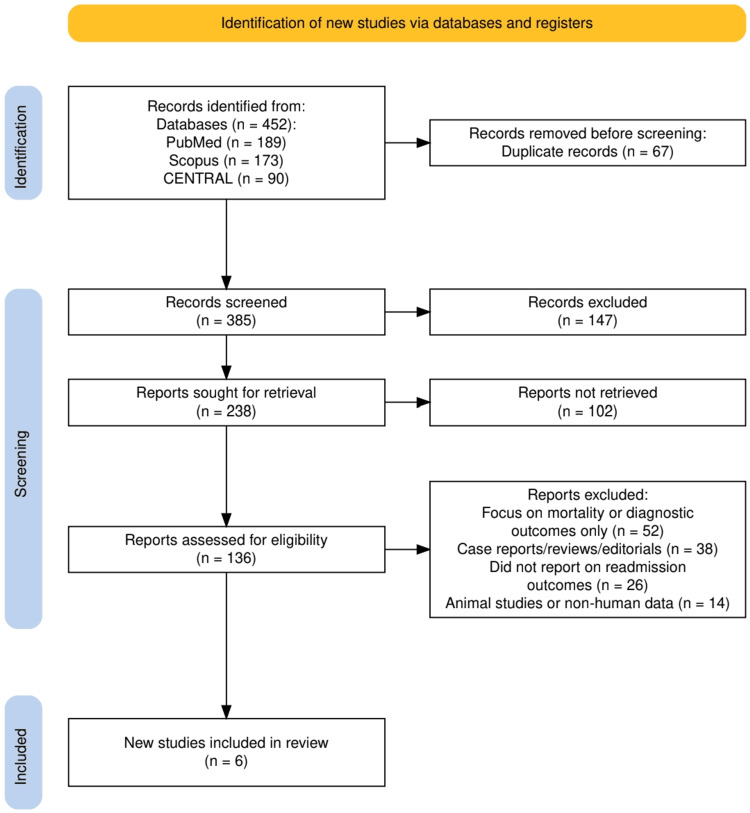
The PRISMA flowchart represents the study selection process. PRISMA, Preferred Reporting Items for Systematic Reviews and Meta-Analyses

The study selection process is summarized in Figure 1, which outlines the identification, screening, and inclusion of studies by PRISMA guidelines. A total of 452 records were initially retrieved through systematic searches across three electronic databases: PubMed (*n* = 189), Scopus (*n* = 173), and CENTRAL (*n* = 90). After the removal of 67 duplicate entries, 385 unique records remained for title and abstract screening. Of these, 147 records were excluded for not meeting the basic relevance criteria. The remaining 238 full-text articles were sought for retrieval, out of which 102 could not be obtained. The remaining 136 full-text reports were assessed for eligibility. Ultimately, 130 studies were excluded based on predefined criteria: focus solely on diagnostic or mortality outcomes (*n* = 52), being case reports, reviews, or editorials (*n* = 38), lacking data on hospital readmissions (*n* = 26), or involving non-human subjects (*n* = 14). As a result, six studies met all inclusion criteria and were included in the final systematic review.

Characteristics of the Selected Studies

The characteristics of the selected studies, as presented in Table 1, reflect a diverse yet focused investigation into the role of NT-proBNP in predicting readmissions among patients with HF. The included studies employed various designs, including randomized controlled trials, post hoc analyses, and both prospective and registry-based observational studies, with sample sizes ranging from under 100 to over 2,000 participants. All studies targeted hospitalized patients with either acute or chronic HF, though there was variation in demographic characteristics, baseline clinical status, and timing of NT-proBNP measurement. Some studies assessed NT-proBNP at a single time point, typically at admission, while others measured it serially throughout hospitalization and into the post-discharge period. Comparators included standard clinical assessment, alternative biomarkers, or gender-based subgroup analyses. Most studies used composite outcomes of all-cause mortality and HF readmission, while a few focused specifically on cardiovascular events or hospital readmission alone. Overall, the studies consistently demonstrated that elevated or persistently high NT-proBNP levels were associated with poorer outcomes, though the strength of association with readmission risk varied depending on the measurement strategy and patient subgroup.

**Table 1:** The summary of the included studies in the review. NT-proBNP, N-terminal pro–B-type natriuretic peptide; HF, heart failure; RCT, randomized controlled trial; PRIMA, pro-brain natriuretic peptide guided therapy of chronic heart failure to improve heart failure morbidity and mortality; BNP, B-type natriuretic peptide; HR, hazard ratio; RDW, red cell distribution width; LV, left ventricular; *P*, *P*-value; mg/mL, milligrams per milliliter

Study (author, year)	Study design	Sample size	Population	NT-proBNP intervention	Comparator (if any)	Primary outcome(s)	Key findings
Stienen et al., 2018 [10]	Randomized Controlled Trial (RCT)	405	Hospitalized patients with acute decompensated HF and NT-proBNP >1700 ng/L	NT-proBNP-guided therapy aiming for >30% reduction from admission to discharge	Conventional treatment without NT-proBNP guidance	Composite of all-cause mortality and HF readmission at 180 days; number of days alive out of hospital in 180 days	More patients in the guided group achieved >30% NT-proBNP reduction (80% vs. 64%, *P* = 0.001); no significant difference in primary or secondary outcomes, including readmission (HR 0.96; *P* = 0.99)
Eurlings et al., 2014 [11]	Post Hoc Analysis of RCT (PRIMA)	309	Patients hospitalized with acute HF	Serial NT-proBNP measurements at admission, discharge, and 1 month post-discharge	No formal comparator group; analysis of changes in NT-proBNP over time	All-cause mortality and the composite of HF readmission or mortality	Change in NT-proBNP during admission, from discharge to 1 month, and absolute level at 1 month independently predicted readmission or death (HRs: 1.71, 2.71, and 1.81; *P* < .05)
Noveanu et al., 2011 [12]	Prospective Multicenter Observational Study	171	Emergency department patients with acute decompensated HF (mean age 80)	Serial NT-proBNP measurements at presentation, 24h, 48h, and discharge	Serial BNP measurements	One-year all-cause mortality (primary); 30-day mortality and one-year HF readmission (secondary)	NT-proBNP and BNP both predicted one-year mortality; prognostic accuracy improved over time; NT-proBNP at discharge predicted mortality (HR 1.07; *P* = 0.016); both biomarkers had poor predictive value for HF readmission.
He et al., 2014 [13]	Prospective Observational Study	128	Hospitalized patients with acute HF	Baseline NT-proBNP measured at admission	Red Cell Distribution Width (RDW)	Cardiovascular events at 30 and 90 days (cardiac death and/or HF readmission)	NT-proBNP was significantly higher in patients with events at 30 and 90 days; NT-proBNP independently predicted 90-day events (HR 3.661; *P* = 0.031); NT-proBNP >1471.5 pg/mL associated with higher risk.
Kim et al., 2017 [14]	Registry-Based Cohort Study	2,280	Patients hospitalized with HF (Korean Heart Failure Registry); 50.9% female	Baseline NT-proBNP levels stratified by tertiles and gender	Gender subgroup analysis (male vs. female)	Composite of all-cause mortality and HF readmission over a mean follow-up of 1,245 days	NT-proBNP was an independent predictor in men (HR 1.74; *P* = 0.001), but not in women (*P* > 0.05)
Fisher et al., 2003 [15]	Prospective Cohort Study	87	Patients admitted with chronic HF due to LV systolic dysfunction.	Baseline NT-proBNP levels measured at admission	Relaxin levels	Death or hospitalization for HF over one year	Patients with NT-proBNP above the median had higher mortality (53%) and readmission/death (70%) vs. the below-median group (11% and 27%, *P* < 0.0001); NT-proBNP was an independent predictor.

Quality Assessment

The quality assessment of the included studies is summarized in Table 2, where each study was evaluated using a design-appropriate appraisal tool to ensure methodological robustness. The randomized controlled trial was assessed with the RoB 2.0 tool [16] and demonstrated a low risk of bias across all domains, indicating strong internal validity. The post hoc analysis of a previous RCT showed overall good methodological quality, though some concerns were noted regarding deviations from intended interventions and selective reporting, due to the secondary nature of the analysis. Observational studies were assessed using the NIH Quality Assessment Tool [17], with one study rated as low risk and another with a moderate risk of bias due to minor concerns in outcome measurement. The two cohort studies were evaluated using the Newcastle-Ottawa Scale [18] and both were found to have a low overall risk of bias, reflecting good quality in selection, comparability, and outcome assessment. Collectively, the quality of the included studies was satisfactory, with most demonstrating low to moderate risk of bias, thereby supporting the reliability of the synthesized findings.

**Table 2:** The quality assessment of each of the selected studies.

Study (author, year)	Study design	Tool used	Randomization process	Deviations from intended interventions	Missing outcome data	Measurement of the outcome	Selection of the reported result	Overall risk of bias
Stienen et al., 2018 [10]	RCT	RoB 2.0	Low risk	Low risk	Low risk	Low risk	Low risk	Low
Eurlings et al., 2014 [11]	Post Hoc of RCT	RoB 2.0 (original RCT) + Reporting appraisal	Low risk (based on parent trial)	Some concerns (post hoc)	Low risk	Low risk	Some concerns	Some concerns
Noveanu et al., 2011 [12]	Observational	NIH Quality Assessment Tool	Not applicable	Low risk	Low risk	Some concerns	Low risk	Moderate
He et al., 2014 [13]	Observational	NIH Quality Assessment Tool	Not applicable	Low risk	Low risk	Low risk	Low risk	Low
Kim et al., 2017 [14]	Registry-Based Cohort	Newcastle-Ottawa Scale (NOS)	Not applicable	Low risk	Low risk	Low risk	Low risk	Low
Fisher et al., 2003 [15]	Prospective Cohort	Newcastle-Ottawa Scale (NOS)	Not applicable	Low risk	Low risk	Low risk	Low risk	Low

Discussion

This systematic review examined the utility of NT-proBNP as a prognostic biomarker for predicting hospital readmissions in patients with HF, synthesizing evidence from six diverse studies encompassing randomized controlled trials, cohort studies, and observational designs. The findings collectively suggest that while NT-proBNP has a well-established role in HF prognosis, its predictive value for readmissions varies depending on how and when it is measured. In the largest and most rigorous randomized controlled trial (RCT) included, the study by Stienen et al. [10], NT-proBNP-guided therapy targeting a >30% reduction during hospitalization did not significantly reduce 180-day readmissions or mortality, despite achieving greater biomarker reduction in the intervention group. This finding contrasts with the post hoc analysis by Eurlings et al. [11], which showed that serial NT-proBNP measurements during admission and especially in the early post-discharge phase were strong, independent predictors of adverse outcomes, including readmission. Similarly, Noveanu et al. [12] demonstrated that while NT-proBNP levels reliably predicted one-year mortality, their ability to predict one-year HF readmission was limited, further emphasizing the distinction between mortality and readmission risk. However, He et al. [13] provided additional evidence that baseline NT-proBNP levels at admission were independently associated with short-term cardiovascular events, including readmissions at 30 and 90 days, particularly when levels exceeded 1471.5 pg/mL. Notably, Kim et al. [14] added a gender-specific nuance by showing that NT-proBNP predicted long-term readmission and mortality in men but not in women, raising questions about the need for sex-specific interpretation. Finally, Fisher et al. [15] found that NT-proBNP levels above the median were strongly associated with higher mortality and readmission within a year, reinforcing its general prognostic value. Collectively, these findings underscore that NT-proBNP is most clinically useful when measured serially, especially during the vulnerable post-discharge window, and that its predictive accuracy may be influenced by timing, threshold values, and patient subgroups such as gender or comorbidities.

The findings of this review are largely consistent with existing literature that recognizes NT-proBNP as a valuable biomarker for risk stratification in patients with HF, particularly in predicting mortality and clinical deterioration [19]. Prior meta-analyses and guidelines, such as those from the ESC and ACC/AHA, have acknowledged the prognostic role of NT-proBNP, especially in identifying patients at higher risk of adverse outcomes [20]. However, our review adds important nuance by focusing specifically on its role in predicting readmissions, an area that has been less thoroughly evaluated. The results from studies like Eurlings et al. [11] and He et al. [13] align with previous research suggesting that dynamic changes in NT-proBNP-rather than single measurements-offer stronger predictive utility for early post-discharge events. This supports the trend toward integrating serial biomarker assessments into discharge planning protocols. On the other hand, the PRIMA II trial by Stienen et al. [10], which failed to show a significant reduction in readmissions despite NT-proBNP-guided therapy, contrasts with smaller studies that reported clinical benefits from biomarker-guided strategies. This discrepancy may reflect differences in study populations, intervention intensity, or thresholds used. Additionally, the gender-specific findings reported by Kim et al. [14] contribute a novel perspective to the literature, which has traditionally applied a one-size-fits-all approach to biomarker interpretation. Overall, our synthesis not only corroborates the broader evidence base but also emphasizes the importance of measurement timing, individualized interpretation, and clinical context when using NT-proBNP to predict HF readmissions.

The variability in NT-proBNP’s ability to predict readmission can be attributed to several physiological and methodological factors. From a physiological standpoint, NT-proBNP levels are influenced not only by cardiac wall stress but also by age, renal function, and body mass index, which may confound their interpretation [19]. Older adults and patients with impaired renal clearance often exhibit elevated baseline NT-proBNP levels unrelated to acute decompensation, potentially reducing its specificity. Methodologically, differences in the timing of NT-proBNP measurement-whether at admission, discharge, or post-discharge-significantly affect its prognostic utility. Studies that employed serial measurements demonstrated better predictive accuracy compared to those relying on a single baseline value. Furthermore, variability in NT-proBNP thresholds across studies and the lack of standardized cut-off values complicate cross-study comparisons and clinical translation [21]. Gender-related differences, as observed in Kim et al., further underscore the need for individualized interpretation of NT-proBNP levels in diverse populations.

This review possesses several strengths that enhance the reliability of its findings. A rigorous, structured literature search was conducted, including only studies that specifically addressed the predictive value of NT-proBNP in HF readmissions. The inclusion of randomized controlled trials, cohort studies, and observational data allowed for a comprehensive understanding of the biomarker’s clinical relevance across varied settings. Furthermore, each study underwent formal quality assessment using design-appropriate tools, improving the methodological transparency of the review. However, several limitations must be acknowledged. The included studies were heterogeneous in terms of patient populations, follow-up duration, timing of NT-proBNP measurement, and defined endpoints. Sample sizes varied widely, and some studies were secondary analyses or post hoc in nature, limiting causal inferences. Additionally, the absence of uniform NT-proBNP thresholds and the frequent use of composite outcomes further complicate the synthesis of results.

The findings of this review have important implications for clinical practice. They highlight the potential of NT-proBNP-particularly when measured serially serve as a useful biomarker for identifying patients at high risk of early readmission following hospitalization for HF [22]. This could facilitate targeted discharge planning, closer outpatient follow-up, and more intensive medical optimization in the post-acute setting. Incorporating NT-proBNP trends into multidisciplinary HF care pathways may enable clinicians to make more informed decisions regarding the timing of discharge and the need for transitional care interventions [23]. However, the results also caution against relying solely on NT-proBNP reduction targets during hospitalization, as evidenced by the neutral findings of the PRIMA II trial [24].

While our review synthesizes findings across diverse study designs, the absence of meta-analysis due to methodological heterogeneity limits the generalizability and quantitative strength of conclusions. Some included studies adjusted for confounders such as age, renal function, and BMI, though this was not uniform, and residual confounding may account for variability in NT-proBNP performance. Additionally, gender-based differences noted in one study may reflect underlying physiological or hormonal influences, though these were not fully explored in the source data. Differences in NT-proBNP thresholds and outcome definitions further limit cross-study comparability, highlighting the need for future standardization to enhance clinical utility.

Despite growing evidence on the prognostic role of NT-proBNP, several gaps remain that warrant further investigation. Future research should focus on standardizing the timing and thresholds for NT-proBNP measurement to improve consistency in clinical application. Large, prospective trials are needed to validate the effectiveness of serial NT-proBNP monitoring as part of structured discharge protocols. Additionally, research should explore the biomarker’s predictive value in specific subgroups, such as patients with renal dysfunction, preserved ejection fraction, or women, in whom NT-proBNP interpretation may differ. Incorporating NT-proBNP into validated risk prediction models, alongside clinical and functional parameters, could further enhance individualized risk assessment and resource allocation in HF management.

## Conclusions

This systematic review demonstrates that NT-proBNP holds substantial potential as a prognostic biomarker for predicting hospital readmissions in patients with HF, particularly when measured serially and interpreted in context. While consistent patterns support its utility, the evidence is not entirely uniform across all study designs or populations. Variability in measurement timing, patient characteristics, and study methodologies may contribute to the differences observed. Although several studies highlight the clinical value of NT-proBNP in discharge planning, limitations such as small sample sizes, post hoc analyses, and methodological heterogeneity should be considered when interpreting the findings. Additionally, practical factors such as the cost and feasibility of serial NT-proBNP measurements may influence its broader implementation in routine care. Further standardized, prospective trials are warranted to establish optimal timing, thresholds, and integration strategies for NT-proBNP in risk-stratification models aimed at reducing hospital readmissions in HF management.

## References

[1] Al-Tamimi MA, Gillani SW, Abd Alhakam ME, Sam KG (2021). Factors associated with hospital readmission of heart failure patients. Front Pharmacol.

[2] Akkineni SSL, Mohammed O, Pathiraj JPK (2020). Readmissions and clinical outcomes in heart failure patients: a retrospective study. Clin Epidemiol Glob Health.

[3] Kripalani S, Theobald CN, Anctil B, Vasilevskis EE (2014). Reducing hospital readmission rates: current strategies and future directions. Annu Rev Med.

[4] Li N, Chen R, Li J (2023). Prognostic significance of serial N-terminal pro-B-type natriuretic peptide levels in patients with acute myocardial infarction: a prospective study. Am Heart J.

[5] Li Y, Xu H, Chen S, Wang J (2024). Advances in electrochemical detection of B-type natriuretic peptide as a heart failure biomarker. Int J Electrochem Sci.

[6] Mouzarou A, Hadjigeorgiou N, Melanarkiti D, Plakomyti TE (2025). The role of NT-proBNP levels in the diagnosis of hypertensive heart disease. Diagnostics (Basel).

[7] Züsli S, Bierreth F, Boesing M (2021). Point of care with serial NT-proBNP measurement in patients with acute decompensated heart failure as a therapy-monitoring during hospitalization (POC-HF): study protocol of a prospective, unblinded, randomized, controlled pilot trial. Contemp Clin Trials Commun.

[8] Brown D (2020). A review of the PubMed PICO tool: using evidence-based practice in health education. Health Promot Pract.

[9] Page MJ, McKenzie JE, Bossuyt PM (2021). The PRISMA 2020 statement: an updated guideline for reporting systematic reviews. BMJ.

[10] Stienen S, Salah K, Moons AH (2018). NT-proBNP (N-terminal pro-B-type natriuretic peptide)-guided therapy in acute decompensated heart failure: Prima II randomized controlled trial (can NT-proBNP-guided therapy during hospital admission for acute decompensated heart failure reduce mortality and readmissions?). Circulation.

[11] Eurlings LW, Sanders-van Wijk S, van Kraaij DJ (2014). Risk stratification with the use of serial N-terminal pro-B-type natriuretic peptide measurements during admission and early after discharge in heart failure patients: post hoc analysis of the PRIMA study. J Card Fail.

[12] Noveanu M, Breidthardt T, Potocki M (2011). Direct comparison of serial B-type natriuretic peptide and NT-proBNP levels for prediction of short- and long-term outcome in acute decompensated heart failure. Crit Care.

[13] He W, Jia J, Chen J (2014). Comparison of prognostic value of red cell distribution width and NT-proBNP for short-term clinical outcomes in acute heart failure patients. Int Heart J.

[14] Kim HL, Kim MA, Choi DJ (2017). Gender difference in the prognostic value of N-terminal pro-B type natriuretic peptide in patients with heart failure: a report from the Korean Heart Failure Registry (KorHF). Circ J.

[15] Fisher C, Berry C, Blue L, Morton JJ, McMurray J (2003). N-terminal pro B type natriuretic peptide, but not the new putative cardiac hormone relaxin, predicts prognosis in patients with chronic heart failure. Heart.

[16] Cochrane Bias Methods Group (2025). RoB 2: a revised Cochrane risk-of-bias tool for randomized trials. https://methods.cochrane.org/bias/resources/rob-2-revised-cochrane-risk-bias-tool-randomized-trials.

[17] De Cassai A, Boscolo A, Zarantonello F (2023). Enhancing study quality assessment: an in-depth review of risk of bias tools for meta-analysis-a comprehensive guide for anesthesiologists. J Anesth Analg Crit Care.

[18] Stang A (2010). Critical evaluation of the Newcastle-Ottawa scale for the assessment of the quality of nonrandomized studies in meta-analyses. Eur J Epidemiol.

[19] Chen C, Hsu YC, Chou KW (2024). NT-proBNP point-of-care testing for predicting mortality in end-stage renal disease: a survival analysis. Heliyon.

[20] Teramoto K, Tay WT, Tromp J (2024). Longitudinal NT-proBNP: associations with echocardiographic changes and outcomes in heart failure. J Am Heart Assoc.

[21] Alzaabi MA, Abdelsalam A, Alhammadi M, Bani Hani H, Almheiri A, Al Matrooshi N, Al Zaman K (2024). Evaluating biomarkers as tools for early detection and prognosis of heart failure: a comprehensive review. Card Fail Rev.

[22] Boesing M, Bierreth F, Abig K (2024). Effects of serial NT-proBNP measurements in patients with acute decompensated heart failure: results of the POC-HF pilot trial. Glob Cardiol Sci Pract.

[23] Berger R, Moertl D, Peter S (2010). N-terminal pro-B-type natriuretic peptide-guided, intensive patient management in addition to multidisciplinary care in chronic heart failure a 3-arm, prospective, randomized pilot study. J Am Coll Cardiol.

[24] Latini R, Masson S (2014). NT-proBNP: a guide to improve the management of patients with heart failure. EJIFCC.

